# Cardiometabolic parameters and novel hematological indices in women with migraine

**DOI:** 10.5937/jomb0-62189

**Published:** 2026-05-22

**Authors:** Aleksandra Klisic, Ana Tmusic

**Affiliations:** 1 University of Montenegro-Faculty of Medicine, Podgorica, Montenegro; 2 Primary Health Care Centre, Podgorica, Montenegro

**Keywords:** coagulation, inflammation, migraine, platelets, koagulacija, inflamacija, migrena, trombociti

## Abstract

**Background:**

The pathophysiological aspect of migraine has not been fully elucidated. It is assumed that inflammation is at the root of this disease. The goal of the research is to examine cardiometabolic and novel haematological indices in patients with migraine.

**Methods:**

A total of 50 women diagnosed with migraine were included in the study. Women were recruited during pain-free periods between pain attacks. The control group consisted of 25 age-matched healthy women. The MIDAS score (Migraine Disability Assessment score) was calculated.

**Results:**

Women with migraine had higher levels of uric acid (P = 0.045) and higher transaminase activity, AST (P&lt; 0.001) and ALT (P= 0.003). Significantly lower neutrophil counts and RDW-CV (%) and higher platelet count (PLT) and PCT were observed in subjects with migraine. The derived haematological parameters, i.e., NLR, dNLR, M-GLR, and PDW/PCT, were significantly lower in the migraine group than in the controls, whereas higher PNR was observed in the migraine group. Among the examined haematological indicators, PNR showed excellent diagnostic accuracy (AUC= 0.727), while other parameters (RDW, PLT, neutrophils, PDW/PCT, NLR, dNLR, M-GLR) showed good diagnostic accuracy (0.600&lt; AUC&lt; 0.700). The largest number of women suffering from migraine (52.9%) had a MIDAS score of 11-20. Creatinine showed good diagnostic accuracy in predicting high MIDAS scores (AUC= 0.678).

**Conclusions:**

Women suffering from migraine have less favourable cardiometabolic status in comparison to healthy women. Blood count parameters and calculated indices have good diagnostic accuracy for migraine. These parameters are cost-effective and easily available.

## Abstract (sr)

**Uvod:**

Patofiziološki aspekt migrene nije do kraja rasvetljen. Pretpostavlja se da se inflamacija nalazi u osnovi ovog oboljenja. Cilj istraživanja je da se ispitaju metabolički i novi hematološki parametri kod pacijenata sa migrenom.

**Metode:**

U istraživanje je regrutovano ukupno 50 žena kod kojih je dijagnostikovana migrena. Ispitanice su regrutovane u bezbolnim periodima, tj. između napada bola. Kontrolnu grupu činilo je 25 zdravih žena, uparenih po godinama starosti. MIDAS skor (engl. Migraine Disability Assessment score) je izračunat.

**Rezultati:**

Žene sa migrenom su imale veći nivo mokraćne kiseline (P= 0,045) i veću aktivnost transaminaza, AST (P&lt; 0,001) i ALT (P= 0,003). Uočen je statistički značajno niži broj neutrofila i RDW-CV (%), kao i veći broj trombocita i vrednost PCT. Kada su u pitanju izvedeni hematološki parametri, NLR, dNLR, M-GLR i PDW/PCT su bili statistički značajno niži u grupi žena obolelih od migrene u poređenju sa pripadnicama kontrolne grupe, dok je PNR bio značajno viši u grupi sa migrenom. Od ispitivanih hematoloških pokazatelja izdvojio se PNR koji je pokazao odličnu dijagnostičku tačnost (AUC= 0,727), dok su ostali parametri (RDW, PLT, neutrofili, PDW/PCT, NLR, dNLR, M-GLR) pokazali dobru dijagnostičku tačnost (0,600&lt; AUC&lt; 0,700). Najveći broj žena koje boluju od migrene (52,9%) je imao vrednost MIDAS skora od 11-20. Kreatinin je pokazao dobru dijagnostičku tačnost u predviđanju visokog MIDAS skora (AUC= 0,678).

**Zaključak:**

Žene koje boluju od migrene imaju nepovoljniji kardiometabolički status od zdravih žena. Parametri krvne slike i parametri izračunati iz njih imaju dobru dijagnostičku tačnost za obolele od migrene, ekonomski su isplativi i lako dostupni.

## Introduction

A migraine is regarded as a recurrent, moderate-to-severe headache that lasts 4-72 hours if left untreated. Apart from extremely intense pain that disables a person from any activity, it is usually accompanied by photophobia, vomiting, nausea, and hypersensitivity to smell and noise [1]. Migraine is a neurovascular disease accompanied by pain and is the sixth most widespread disorder in the world and the main driving force of disability, which represents a significant burden for individuals and the community [2]. About 15.1% of the general population suffers from this type of headache [2][3]. Studies have shown that migraine is dominant in individuals between 30 and 39 years of age and that women have a higher incidence rate than men, regardless of age [4].

Migraines significantly affect the quality of life and success at the workplace, as well as unemployment, which is higher among people who are treated for migraine [1]. About 75% of them have diminished workplace functioning, and more than 50% are absent from work. Usually, migraine attacks are sporadic. However, some people experience a steady rise in frequency of attacks over time, and in up to 2% of the overall population, migraine progresses to chronicity. Chronic migraine is regarded as a headache lasting ≥15 days/month for ≥3 months, of which there are at least eight migraine days/month for ≥three months [1][2][3][4].

In addition to genetic factors, fluctuations in the level of sex hormones (progesterone and estrogen) and external factors can cause migraine. Triggers can vary from person to person, but the most frequently described triggers include stress, lack of sleep, menstruation, (sun)light, certain foods, skipping meals, alcoholic beverages, mild head trauma, weather changes, and high altitudes. However, the exact mechanisms of these triggering factors have not yet been elucidated [1][2][3][4]. Indeed, even though migraine is a neurological disease known for its long duration, the pathophysiological mechanisms are still unclear [3]. It is assumed that this disorder is based on »sterile inflammation,« i.e., neurogenic neuroinflammation [2][3]. Classical neurogenic inflammation in peripheral tissues is initiated by the secretion of substances such as substance P neurokinin A, and calcitonin gene-related peptide (CGRP) from the peripheral endings of peptide-containing sensory nerve fibres. This process includes white blood cell recruitment, plasma extravasation, vasodilation, and mast cell degranulation [3][5]. Activated mast cells generate proinflammatory cytokines/chemokines that precede migraine and neurological symptoms, such as nausea, fatigue, and brain fog [6][7][8][9].

Recently, several studies have examined the relationship between blood count parameters and inflammation across various pathological conditions. Namely, the parameters of the complete or differential blood count, and their indices, are attracting attention [10]. Previous studies have shown a higher level of leukocytes with a simultaneously higher level of neutrophils in obese people and people with type 2 diabetes [11][12]. A few studies focused on new indices derived from leukocyte subpopulations in various chronic diseases and demonstrated their superiority relative to leukocyte counts and their indices [13][14]. The physiological response of leukocytes to stressful conditions, such as inflammation, leads to an increase in neutrophil count and a decrease in lymphocyte count. The ratio of these 2 leukocyte subpopulations is also used as an index of inflammation [15][16][17][18][19]. Monocytes are a mononuclear subpopulation of leukocytes that play important roles in both acquired and innate immunity. The recruitment of monocytes into tissues is a significant feature of inflammation [20][21][22][23].

Several meta-analyses described the association between cardiovascular disease (CVD) and migraine [24]. Migraines are also known to be related to cardiovascular risk factors, including obesity and type 2 diabetes. Individuals with migraine, compared to those without such a disorder, have higher rates of hypercholesterolemia and hypertension [24]. The underlying pathophysiological mechanisms linking migraine and CVD may include limited vasodilation, the dysfunction of endothelium, uncontrolled platelet activation, platelet aggregation, and the use of nonsteroidal anti-inflammatory medications [25]. However, the pathophysiological mechanisms that link migraine with CVD have not been fully elucidated. The goals of this research were to examine cardio-metabolic and novel haematological (calculated/derived from basic, routine haematological analyses) parameters in women with migraine.

## Materials and methods

### Patients

A total of 50 women who were diagnosed with migraine [International Classification of Diseases (ICD-10), code G43] and who were regularly monitored at the Primary Health Centre, Podgorica, Montenegro, were included in the research. Subjects were recruited during pain-free periods between pain attacks. The control group consisted of 25 healthy age-matched individuals.

Recruitment of subjects, blood sampling, biochemical analyses, and clinical check-ups were done at the Primary Health Care Centre - Centre for Laboratory Diagnostics, Podgorica, Montenegro, after the Ethics Committee of the said institution approved the study protocol.

The research was conducted in accordance with the ethical standards of the Declaration of Helsinki. The participants were informed about the aim of the research, the methods to be used, and that they could withdraw from the research at any time. All respondents signed an informed consent to participate in the research.

The inclusion criteria in the study were as follows: women aged 20-50 who, apart from migraine, had no other diseases.

Exclusion criteria: other neurological disorders, mental illnesses, metabolic disorders (diabetes, thyroid disorders, gout), liver and kidney diseases, neoplastic and autoimmune diseases, pregnancy, breastfeeding, and menopause.

### Methods

Arterial blood pressure was measured for each individual, body height and weight were measured, while body mass index (BMI) was calculated.

### MIDAS score

The MIDAS questionnaire [Migraine Disability Assessment (MIDAS) score] for the assessment of disability caused by migraine was administered to each subject suffering from migraine in order to assess the influence of headaches on the quality of life [26]. Score results represent a physician's independent assessment of disability, pain, and treatment needs, based on the patient's medical history, and range from 0 (lowest) to 100 (highest) in disability.

MIDAS score categories:<br>I - No or slight disability 0-5<br>II - Mild disability 6-10<br>III - Moderate disability 11-20<br>IV - Severe disability 21+

### Biochemical and haematological analyses

Blood sampling was performed in the morning, after an overnight fast of at least 8 hours. Blood samples were collected in tubes containing K_2_EDTA (for complete blood count from whole blood) and in tubes containing a clot activator and a serum separator.

Whole blood samples were analysed immediately after sampling on an automated haematology counter, the Sysmex XN-1000 analyser (Sysmex Corporation, Kobe, Japan), for erythrocytes, platelets, leukocytes, and their subpopulations as part of the complete blood count.

Samples in tubes containing clot activator and serum separator were left to stand for 30 min after coagulation was complete, then centrifuged at 3000 rpm for 10 min to isolate the serum. The obtained serum samples after centrifugation were used for the analysis of the following parameters: glucose, lipids [i.e., total cholesterol, triglycerides (TG), high-density lipoprotein cholesterol (HDL-c), low-density lipoprotein cholesterol (LDL-c)], high-sensitivity C-reactive protein (hsCRP), uric acid, creatinine, gamma-glutamyl transferase (GGT), alanine aminotransferase (ALT), and aspartate aminotransferase (AST).

All biochemical parameters were analysed on a Roche Cobas c503 biochemical analyser (Roche Diagnostics GmbH, Mannheim, Germany). In addition to the traditional markers of inflammation, the total number of leukocytes, neutrophils, and lymphocytes, their indices were calculated as novel parameters of inflammation: the ratio of monocytes/granulocytes and lymphocytes [M/GLR = (leukocytes-lymphocytes)/lymphocytes], neutrophil/lymphocyte ratio (NlR), derived NLR [dNLR = neutrophils/(leukocytes-neutrophils)], platelet/lymphocyte ratio (PLR), platelet/neutrophil ratio (PNR), lymphocyte/monocyte ratio (LMR), and neutrophil/monocyte ratio (NMR).

### Statistical analysis

The data distribution was checked using the Shapiro-Wilk test (for groups of up to 50 subjects). The Mann-Whitney U test was used to compare differences between two groups of unrelated data, while the Kruskal-Wallis test was used to compare 3 or more groups of data. Receiver operating characteristic (ROC) analysis was used to assess the diagnostic accuracy of migraine assessment in test subjects.

Multiple linear regression analysis of predictors of the MIDAS score, as a clinical indicator of patient status, was applied to determine the best model for predicting this parameter. For all statistical tests, the basic criterion for statistical significance is that the obtained P in a two-sided test be ≤0.05 (specified level of significance α).

## Results

The subjects of this study were younger women, of reproductive age (n = 50), with an average age of about 40 years, BMI <25 kg/m^2^ and an average MIDAS score of about 12 (Table 1). Healthy test subjects had a statistically significantly higher glucose concentration than those with migraine. Given that these values for both examined groups were within the reference range, further analysis of these values and observed differences has no clinical significance. The activities of enzymes that indicate liver function, ALT and AST, were statistically significantly higher in the group of female patients compared to healthy individuals, although these activities are also within the reference range. An interesting result is the significantly higher concentration of uric acid in the group of patients with migraine, because it is considered that this disease basically has an inflammatory component.

**Table 1:** Basic clinical and biochemical parameters in the group of women with migraine and the control group of healthy individuals.

Parameter	Control group (n = 25)	Group with migraine (n = 50)	P
Age (years)	43 (42-44)	40 (34-46)	0.055
MIDAS score	/	12 (9-17)	/
BMI (kg/m^2^)	24.5 (23.2-25.7)	23.3 (21.5-25.3)	0.072
Glucose (mmoL/L)	5.40 (5.10-5.60)	5.00 (4.75-5.25)	0.006
hsCRP (mg/L)	0.880 (0.405-1.135)	0.900(0.400-1.650)	0.414
Creatinine (μmol/L)	63 (55-68)	62 (58-70)	0.500
Uric acid (μmol/L)	201 (187-222)	220 (190-260)	0.045
GGT (IU/L)	11 (9-15)	12 (9-14)	0.824
AST (U/L)	18 (16-21)	23 (20-27)	<0.001
ALT (U/L)	19 (14-22)	23 (18-30)	0.003
Total cholesterol (mmol/L)	5.21 (4.59-5.68)	5.09 (4.76-5.76)	0.987
HDL-c (mmol/L)	1.64 (1.22-1.84)	1.70 (1.46-1.98)	0.340
LDL-c (mmol/L)	3.07 (2.33-3.55)	2.94 (2.51-3.63)	0.637
TG (mmol/L)	0.980 (0.765-1.205)	1.060 (0.735-1.295)	0.691
non-HDL-c (mmol/L)	3.50 (2.82-4.02)	3.41 (2.97-4.26)	0.558
TG/HDL-c ratio	0.61 (0.45-1.03)	0.60 (0.43-0.92)	0.625

The MIDAS score in the migraine group ranged from 3 to 30. The largest number of patients had a MIDAS score of 11-20 (52.9%) (Table 2).

**Table 2:** Clinical status of persons suffering from migraine assessed using the MIDAS score.

MIDAS score, n (%)	0-5	6-10	11-20	>21
	9 (17.6)	10 (19.6)	27 (52.9)	5 (9.8)
	Minimum	Maximum	Mean value	Standard deviation
Basic statistical parameters	3.0	30.0	12.0	6.2
Median (25. -75. percentiles)	12 (9-18)			

Table 3 shows basic and derived (calculated) haematological parameters. There is a significant difference in neutrophil count (significantly lower in patients with migraine) and in RDW-CV (%). On the other hand, the platelet count is significantly higher in patients with migraine, as is the PCT value. NLR, dNLR, M-GLR, and PDW/PCT were significantly lower in the migraine group of women than in the control group, whereas PNR was significantly higher in the migraine group.

**Table 3:** Haematological parameters in migraine sufferers and healthy women. Legend: NLR=Neu/Lymph; dNLR = Neu/ (WBC-Neu); M-GLR=(WBC-Lymph)/Lymph; PLR = PLT/Lymph; PNR = PLT/Neu; MPV/PLT; PDW/PCT

Parameter	Control group (n = 25)	Group with migraine (n=50)	P
WBC (×10^9^/L)	6.69 (5.63-7.86)	6.37 (5.59-7.21)	0.276
Neu (×10^9^/L)	3.79 (2.78-4.80)	3.00 (2.53-3.79)	0.017
Lymph (×10^9^/L)	2.13 (1.61-2.56)	2.37 (2.01-2.77)	0.151
RBC (×10^12^/L)	4.49 (4.26-4.71)	4.13 (3.94-4.34)	0.102
Haemoglobin (g/L)	128 (121-138)	125 (120-131)	0.256
Hematocrit (L/L)	0.389 (0.370-0.417)	0.376 (0.358-0.400)	0.623
RDW-CV (%)	13.0 (12.2-14.3)	13.0 (12.2-14.3)	0.023
Platelets (×10^9^/L)	251 (200-283)	291 (254-325)	0.034
PDW (fL)	11.4 (9.7-13.0)	11 (10-12)	0.573
MPV (fL)	10.0 (9.2-10.6)	10.0 (9.5-10.7)	0.642
PCT (L/L)	0.0024 (0.0021-0.0029)	0.0029 (0.0026-0.0032)	0.001
NLR	1.76 (1.23-2.50)	1.36 (0.99-1.73)	0.018
dNLR	1.29 (0.94-1.86)	0.99 (0.75-1.30)	0.009
M-GLR	2.05 (2.05-2.80)	1.75 (1.24-2.08)	0.028
PLR	118 (94-141)	116 (91-160)	0.864
PNR	67 (46-87)	90 (69-120)	0.001
MPV/PLT	0.039 (0.033-0.053)	0.036 (0.030-0.042)	0.128
PDW/PCT	4458 (3824-6794)	3891 (3344-4592)	0.033

We divided the group of patients with migraine into three subgroups based on hsCRP terciles (Table 4). Total cholesterol, LDL-c and non-HDL-c were statistically significantly higher in subgroups with the highest hsCRP concentrations. There was no difference in the MIDAS score across levels of hsCRP concentration.

**Table 4:** Relationship between biochemical parameters and the level of subclinical inflammation.

Parametar	hsCRP terciles (mg/L)			P
	I (<0.50)	II (0.50-1.30)	III (>1.30)	
MIDAS score	12 (9-18)	11.5 (6-16.5)	12 (9-13.5)	0.937
Age (years)	37 (32-42)	40 (33-46)	43 (39-47)*	0.095
BMI (kg/m^2^)	21.6 (20.5-23.3)	24.3 (23.0-25.4)	23.6 (22.8-26.8)*	0.102
Uric acid (μmol/L)	213 (169-247)	217 (167-243)	252 (209-298)*,#	0.064
Total cholesterol (mmol/L)	4.81 (4.44-5.16)	5.29 (4.88-6.03)*	5.38 (4.88-6.28)*	0.026
LDL-c (mmol/L)	2.69 (2.18-3.13)	2.99 (2.67-3.36)	3.35 (2.78-3.95)*	0.045
TG (mmol/L)	0.88 (0.69-1.12)	0.86 (0.67-1.95)	1.16 (1.06-1.56)**	0.058
Neu (×109/L)	2.76 (2.24-3.32)	2.99 (2.61-3.61)	1.16 (1.06-1.56)**	0.089
TG/HDL-c ratio	0.48 (0.36-0.67)	0.58 (0.37-1.21)	0.75 (0.59-1.07)*	0.119
non-HDL-c (mmol/L)	3.10 (2.52-3.49)	3.35 (3.03-4.33)	3.70 (3.34-4.66)*	0.030

**Figure 1:**
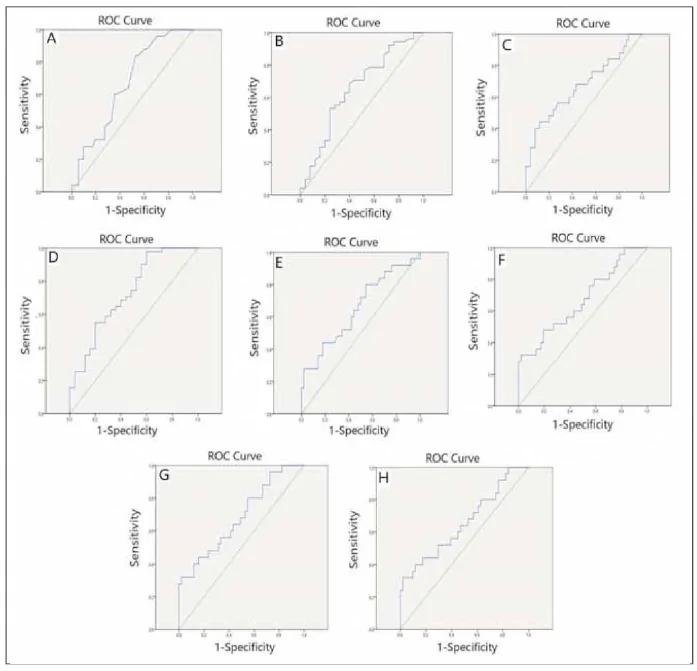
ROC curves for the following parameters measured in this study: A. RDW; B. PLT; C. PNR; D. NEU; E. PDW/PCT; F NLR; G. dNLR; H. M-GLR.<br>Legend: PNR=PLT/Neu; NLR=Neu/Lymph; dNLR=Neu/ (WBC-Neu); M-GLR=(WBC-Lymph)/Lymph; MPV/PLT

ROC analysis was applied to examine the diagnostic potential of new parameters, calculated (derived from basic, routine haematological analyses), in predicting migraine. The results are presented in Figure 1 and in Table 5.

**Table 5:** ROC curve analysis for testing the diagnostic accuracy of the parameters measured in the study in women suffering from migraine. AUC - area under the curve, SE - standard error. Legend: NLR=Neu/Lymph; dNLR=Neu/(WBC-Neu); M-GLR=(WBC-Lymph)/Lymph; PLR=PLT/Lymph; PNR=PLT/Neu; MPV/PLT; PDW/PCT

Parameters	AUC (95% CI)	SE	P
RDW-CV (%)	0.662 (0.539-0.784)	0.063	0.023
PLT (×10^9^/L)	0.651 (0.515-0.786)	0.069	0.034
Neu (×10^9^/L)	0.670 (0.533-0.806)	0.070	0.017
PNR (PLT/Neu)	0.727 (0.603-0.851)	0.063	0.001
PDW/PCT	0.652 (0.516-0.788)	0.069	0.033
NLR (Neu/Lymph)	0.667 (0.534-0.800)	0.068	0.018
dNLR (Neu/(WBC-Neu)	0.685 (0.557-0.814)	0.066	0.009
M-GLR (WBC-Lymph)/Lymph	0.656 (0.519-0.792)	0.070	0.028

The parameters shown in Table 5 (haematological parameters and parameters calculated from them) demonstrated statistically significant diagnostic accuracy; only one parameter (PNR, AUC>0.700) showed excellent diagnostic accuracy, and all others showed good diagnostic accuracy (AUC 0.600 but<0.700).

The following ROC analysis was performed to determine whether some of the parameters identified in this study have good diagnostic accuracy for predicting a high MIDAS score (MIDAS ≥11 points), corresponding to a more severe clinical stage of migraine.

**Figure 2:**
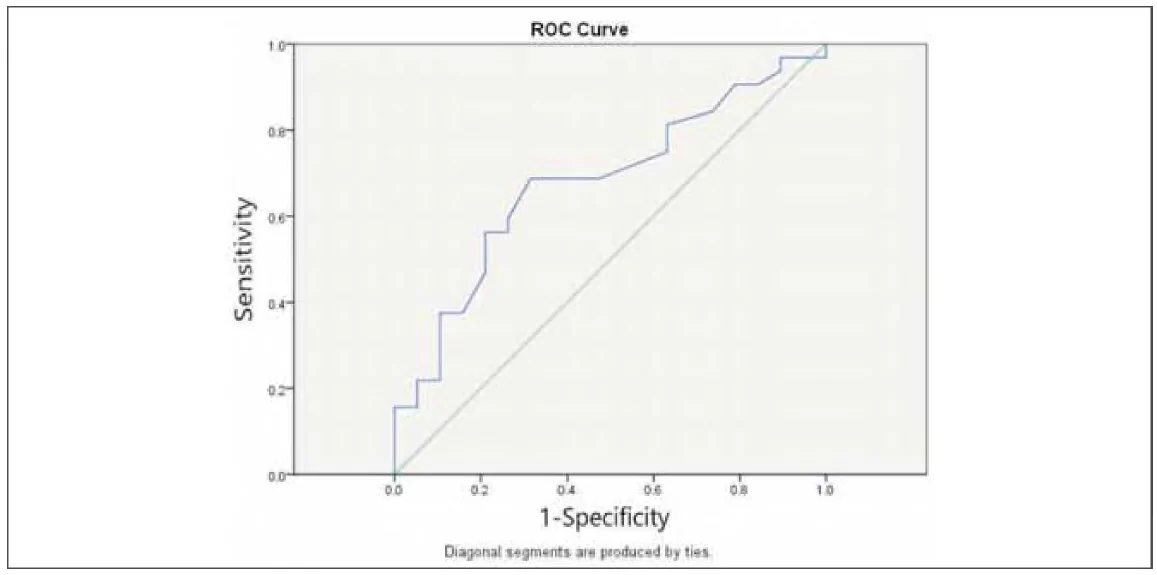
ROC curve for predicting high MIDAS score values (≥11)

The only parameter with significant diagnostic accuracy for predicting a high MIDAS score was creatinine (Figure 2, Table 6), although its AUC was below 0.700.

**Table 6:** ROC analysis in the prediction of high MIDAS score values (≥11 points) in women with migraine.

Parameter	AUC (95% CI)	SE	P
Creatinine	0.678	0.076	0.035

Multiple linear regression was performed to assess the influence of the determined parameters on the MIDAS score. The results are shown in Table 7.

**Table 7:** Multiple linear regression analysis for defining predictors of MIDAS score values.

Predictors of the MIDAS score<br>(adj. R^2^=0.113)	B (95% CI)	SE	P
Creatinine	0.210 (0.027-0.392)	0.091	0.026
DBP	0.488 (0.008-0.968)	0.238	0.046

The initial predictors included in the analysis were: DBF, RBC, HDL-c, LDL-c, creatinine, leukocyte count, platelet count, hsCRP and TG. The best model consisted of the parameters creatinine and DBF. The strongest predictor in the model was creatinine, followed by DBP.

## Discussion

The aforementioned research was conducted to examine cardiometabolic parameters and novel haematological indices in women with migraine. The results show a significant difference in cardio-metabolic risk factors in women with migraine compared to healthy women. Namely, women with migraine had higher levels of uric acid and higher transaminase (AST and ALT) activity, while lipid status parameters did not differ between the studied groups.

hsCRF is a sensitive biomarker of active systemic inflammation that independently correlates with cardiovascular morbidity [3]. In our research, we did not find a difference in hsCRF concentration between the study and control groups. We assume that one reason is the small number of respondents in the research. However, regarding the hypothesis of a connection between subclinical chronic inflammation and the development of migraine [3], we divided the migraine patient group into three subgroups based on hsCRP tertiles. Total cholesterol, non-HDL-c, and LDL-c were significantly higher in the subgroups with the highest hsCRP concentrations, indicating a clear relationship between inflammation and dyslipidemia as CVD risk factors in women with migraine [26].

In our research, we observed lower neutrophil counts and lower RDW-CV in patients with migraine. On the other hand, the platelet (PLT) count was higher in subjects with migraine, as was the PCT value. Among the derived haematological parameters, NLR, dNLR, M-GLR, and PDW/PCT were lower in women with migraine than in the control group, whereas PNR was higher in the migraine group. The obtained results indicate that migraine should be assumed as an important risk factor for CVD. The total leukocyte count (WBC), the number of subtypes (e.g., lymphocytes and neutrophils), and their ratios can also serve as markers of systemic inflammation [27][28].Leukocytes are involved in the immune response, whereas platelets are primarily considered mediators of thrombosis and hemostasis. The inflammatory immune response is associated with platelet adhesion and activation of the coagulation cascade (27). Platelets interact with leukocytes at the site of vascular injury, leading to vascular inflammation, thrombosis, and hemostasis [27].

Also, Komürcü et al. [29] confirmed lower RDW (i.e., an indicator of erythrocyte size and volume) and higher PLT count in pain-free periods between pain attacks in individuals with episodic migraine without aura.

Zeller et al. [30] reported increased platelet activation and leukocyte-platelet aggregates in patients with migraine without aura during the attack-free period, compared with healthy subjects. This finding supports the hypothesis that chronic inflammation and hemoconcentration are persistent even in the absence of pain attacks in patients with migraine without aura.

Platelets interact with leukocytes, forming aggregates and bridges between leukocytes and the endothelium. Platelet P-selectin mainly moderates this process. Through their interactions with monocytes, lymphocytes, neutrophils, and endothelium, platelets are major drivers of inflammation and innate and acquired immune responses [3]. The link between the pathogenesis of migraine and the number of platelets is in accordance with earlier research indicating that antiplatelet drugs are effective in reducing the severity of migraine pain [31].

Research shows that platelet function abnormalities in individuals with migraine can lead to platelet abnormalities, particularly changes in platelet membrane viscosity. Oxman et al. [32] analysed the levels of cholesterol and phospholipids in platelet membranes in individuals with migraine. In comparison with the control group, they confirmed a significant increase in phosphatidylcholine and arachidonic acid in individuals with frequent migraine attacks, as well as in subjects without frequent attacks of migraine [32].

The link between migraine and CVD was demonstrated in a representative sample of 5,692 adult respondents in the USA [25]. It is assumed that a common pathophysiological mechanism underlies these two diseases. The key points closely related to the pathogenesis of both migraine and CVD include endothelial dysfunction, platelet aggregation, vasoconstriction, and coagulation [25].

In our research, ROC analysis was used to assess the diagnostic potential of new parameters, derived from basic, routine haematological analyses, for predicting migraine. Haematological parameters and parameters calculated from them showed significant diagnostic accuracy. Of all haematological indicators, PNR showed excellent diagnostic accuracy (AUC > 0.700), while the other parameters (RDW, PLT, neutrophils, PDW/PCT, NLR, dNLR, M-GLR) showed good diagnostic accuracy (0.600<AUC <0.700). PNR is associated with inflammation and thrombosis, as activated platelets secrete mediators that recruit leukocytes. Platelets interact with neutrophils and leukocytes, exacerbating thrombosis and inflammation [27].

Women suffering from migraine exhibit higher activity of transaminases (i.e., AST and ALT) as compared with healthy counterparts. This finding may not be a direct relationship between transaminases and migraine, but it could rather be attributed to the use of medications that are metabolised via the liver and that exert hepatotoxic effects [33][34]. The other possible explanation is the relationship between migraine and other comorbidities (e.g., obesity, metabolic syndrome, and diabetes) that are often accompanied by higher activity of liver enzymes [24].

It is assumed that oxidative stress and inflammation are underlying features of migraine. Women with migraine in our study exhibited higher levels of uric acid in comparison with the control group, which can also be attributed to potential comorbidities in women with migraine [24]. Our findings are the opposite of those of Yang et al. [35], who found lower levels of uric acid and creatinine, which may explain its antioxidative effects in migraine.

The largest number of women suffering from migraine (52.9%) had a MIDAS score of 11-20, which speaks in favour of the fact that most of these patients have moderate disability. Multiple linear regression was performed to assess the influence of the determined parameters on the MIDAS score. The strongest predictor in the model was creatinine, followed by DBF. ROC analysis was performed to determine whether the parameters identified in this study have good diagnostic accuracy for predicting a high MIDAS score (MIDAS 211 points). The only parameter that showed significant diagnostic accuracy in predicting a high MIDAS score was creatinine (AUC = 0.678). The link between serum creatinine level and MIDAS score may not be direct but is confounded by the potential cumulative effects of medications [33], since women with higher MIDAS scores are more likely to be on medication treatment than those with lower MIDAS scores. The other possible explanation might be attributed to potential comorbidities that can affect renal function, such as hypertension. Indeed, recent research indicates a connection between migraine and hypertension, primarily with DBP [36]. It is hypothesised that the mechanism by which DBP is a contributor to migraine may include chronic damage to the endothelium, which requires further investigation. The association between DBP and migraine has uncovered several potential regulatory pathways, predominantly the renin-angiotensin system, cardiovascular system, calcium signalling pathways, and metabolic pathways [36].

The aforementioned research has certain limitations, including the lack of investigation into cardiometabolic biomarkers during migraine attacks. Also, we cannot conclude that migraine causes the investigated parameters, given that the aforementioned study was cross-sectional. Longitudinal studies with larger sample sizes of both genders are necessary to elucidate the various pathophysiological aspects of migraine.

## Conclusions

Women suffering from migraine have less favourable cardiometabolic status in comparison to healthy women. Blood count parameters and calculated indices have good diagnostic accuracy for migraine. These parameters are cost-effective and easily available and can serve as a reliable diagnostic tool in women with this disorder.

## Dodatak

### Conflict of interest statement

All the authors declare that they have no conflict of interest in this work.
